# Retinal Autophagy for Sustaining Retinal Integrity as a Proof of Concept for Age-Related Macular Degeneration

**DOI:** 10.3390/ijms26125773

**Published:** 2025-06-16

**Authors:** Roberto Pinelli, Gloria Lazzeri, Caterina Berti, Francesca Biagioni, Elena Scaffidi, Michela Ferrucci, Violet Vakunseh Bumah, Francesco Fornai

**Affiliations:** 1 SERI, Switzerland Eye Research Institute, 6900 Lugano, Switzerland; roberto@seri-lugano.ch (R.P.); backoffice@seri-lugano.ch (C.B.); elena@seri-lugano.ch (E.S.); 2 Human Anatomy, Department of Translational Research and New Technologies in Medicine and Surgery, University of Pisa, 56126 Pisa, PI, Italy; gloria.lazzeri@unipi.it (G.L.); michela.ferrucci@unipi.it (M.F.); 3 IRCCS Neuromed, 86077 Pozzilli, IS, Italy; francesca.biagioni@neuromed.it; 4 Department of Chemistry and Biochemistry Bush Mathematical Sciences Building Suite 108, Stephen F. Austin State University, Nacogdoches, TX 75962, USA; vbumah@sdsu.edu; 5 Department of Chemistry and Biochemistry, College of Sciences, San Diego State University, 5500 Campanile Drive, San Diego, CA 92182, USA

**Keywords:** retinal degeneration, retinal pigment epithelium, proteasome, lysosome, autophagoproteasome, lipophagy, mitophagy, drusen, pseudodrusen, retinal neurovascular unit

## Abstract

Current evidence indicates that most types of autophagy represent a pivot in promoting retinal integrity. In healthy conditions, autophagy acts on multiple pathways, which are fundamental for the biochemistry and the fine structure of the retina. Autophagy is essential in granting visual processes. On the other hand, autophagy dysfunction characterizes several retinal disorders. This is mostly evident in age-related macular degeneration (AMD), which represents the most common degenerative disease leading to blindness. The involvement of autophagy in AMD is documented in vitro and in vivo experiments, and it is strongly suggested by clinical findings in humans. The present manuscript provides an overview of the specific types of autophagy, which prevail in the retina and their alterations in retinal degeneration with an emphasis on AMD. The dysfunction of specific autophagy steps was analyzed in relation to hallmarks of AMD pathology and symptoms. An extended session of the manuscript analyzes the connection between altered autophagy and cell pathology within retinal pigment epithelium, as well as the site and structure of extracellular aggregates named drusen. The significance of the drusen in relation to visual function is discussed in the light of the role of autophagy in regulating key steps of phototransduction.

## 1. General Introduction

**Figure 1:**
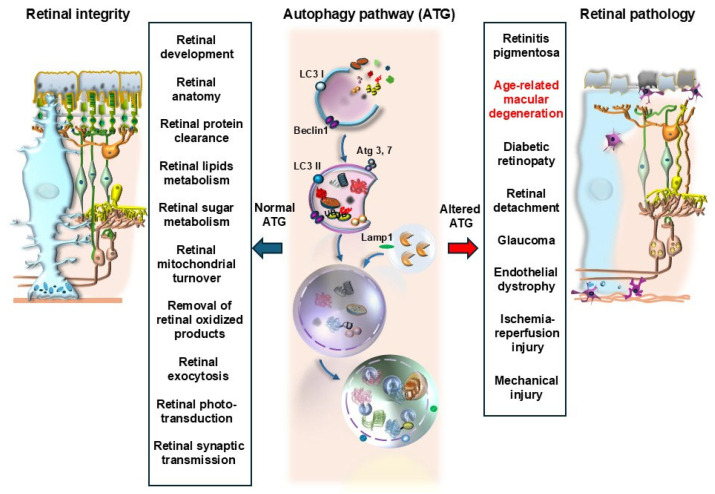
Introductory graphical scheme of various types of autophagy and various retinal disorders. The concept of autophagy as a relevant pathway in the retina is rather generic since multiple types of autophagy exist, and their role may vary depending on the specific retinal layer and disorders. The cartoon reporting the prototype of autophagy vacuoles is also drawn according to a general scheme, which includes the removal of various chemical species (proteins, lipids, carbohydrates, nucleic acids) and organelles (mitochondria, endoplasmic reticulum). Within specific retinal disorders, which are featured by altered autophagy, a difference may exist concerning specific types of autophagy. It is likely that the occurrence of retinal damage following various pathological conditions of the eye may be produced by a defect in distinct autophagy steps. The effort to distinguish between various forms of autophagy and to dissect which kind is most relevant in AMD represents a major aim of the present manuscript.

The clearing system, named autophagy, plays a key role in sustaining the integrity of the mammalian retina and has vast implications concerning the modulation of various disorders leading to retinal degeneration (Figure 1). When digitizing the keywords “autophagy and retina” and carrying out a PubMed search, the number of articles published in 2005 corresponds to zero papers, while this search sorts two documents in 2006, showing over 100 manuscripts per year during the last five years. Thus, the available literature on the topic covers roughly 1000 manuscripts, which were published at an increasing rate, in less than two decades.

**Figure 2:**
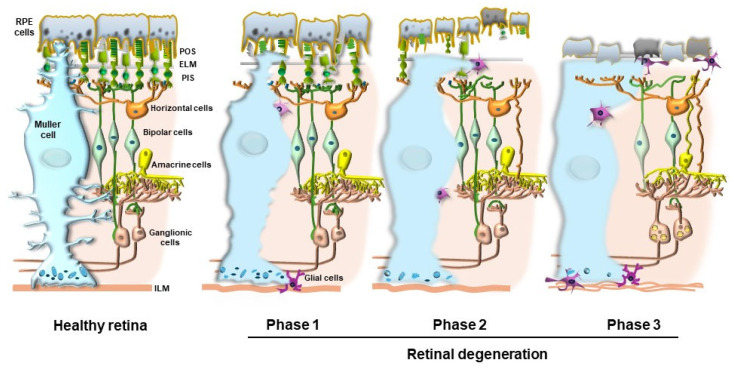
Autophagy operates within a healthy retina, and its alteration occurs at late stages of most retinal degenerative disorders. Retinal degenerative disorders featuring early loss of photoreceptors consist of several forms of retinal degenerative diseases. In these disorders, which start at the level of the outer retina, where RPE and the outer segment of the photoreceptor are placed, a progression downstream to the inner retina often occurs according to a common convergent pathology. This recruits various cell layers of the retinal circuitry to reach the ganglionic cell layer, as indicated in the figure labelling. This produces retinal maladaptive plasticity [23], where alterations of retinal structure are bound to autophagy failure [9,16,23]. At the final stage, the widespread retinal pathology mimics central neurodegenerative disorders [23,24,25]. ELM = external limiting membrane; ILM = inner limiting membrane; PIS = photoreceptor inner segment; POS = photoreceptor outer segment.

The impressive increase in reports dealing with retinal autophagy concerns both pre-clinical research and clinical findings. This provides solid evidence about how hot the topic is in current research, which fully justifies the focus on autophagy in sustaining retinal integrity as the object of the present special issue. An increasing awareness exists about the relevance of dysfunctional autophagy in the general context of retinal degeneration and within specific retinal disorders [1]. Multiple retinal diseases are connected with dysfunctional autophagy, although this is mostly relevant considering degenerative disorders. Thus, the study of autophagy within degenerating retina clusters has led to intense translational research moving from basic and preclinical experimental studies [2,3,4,5,6,7], which are in line with clinical findings [8,9,10,11,12,13,14]. Among multiple degenerative conditions of the retina, most reports indicate a fundamental role of autophagy in the course of age-related macular degeneration (AMD) [15]. This is evident both from experimental models and from reports obtained in studies from human subjects [8,10,11,12,16,17,18]. Since AMD prevails among various kinds of retinal degeneration, and it represents the most common cause of blindness in industrialized countries, the present manuscript emphasizes the role of autophagy in sustaining retinal integrity and its failure as a central trigger of AMD. Nonetheless, one should not neglect that the role of autophagy extends beyond the border of AMD, embracing most retinal degenerative disorders. For instance, autophagy modulates the course of *Retinitis pigmentosa* (RP) [19,20], and autophagy dysfunction within altered neuronal and endothelial retinal cells is involved in diabetic retinopathy [21]. Again, recent evidence indicates an important role of autophagy in the degeneration of retinal ganglion cells, which happens during ischemia-reperfusion injury, mechanical injury, and glaucoma [22] (Figure 1). Finally, despite featuring various syndromes owing to a distinct early pathology, most types of retinal degeneration at late stages converge toward a massive derangement of retinal structure, where autophagy failure is a leading mechanism [23]. In the course of this, convergent late-stage retinal degeneration occurs as a spreading phenomenon from the outer to the inner retina under the strong modulation of the autophagy machinery [9,16,23] (Figure 2). At this stage, the widespread retinal degeneration recapitulates the final stages of central neurodegenerative disorders characterized by altered cell clearance [23,24,25].

Therefore, altered autophagy is suggestive of late disease progression, which takes place also in several classic neurodegenerative disorders outside the retina. In the last part of the present manuscript, the suggestive hypothesis that interconnected steps involved in AMD may recapitulate basic mechanisms of degeneration within the CNS will be discussed, including trans-synaptic spreading among various neuronal phenotypes of the retina (Figure 2).

## 2. Introducing Autophagy in AMD

**Figure 3:**
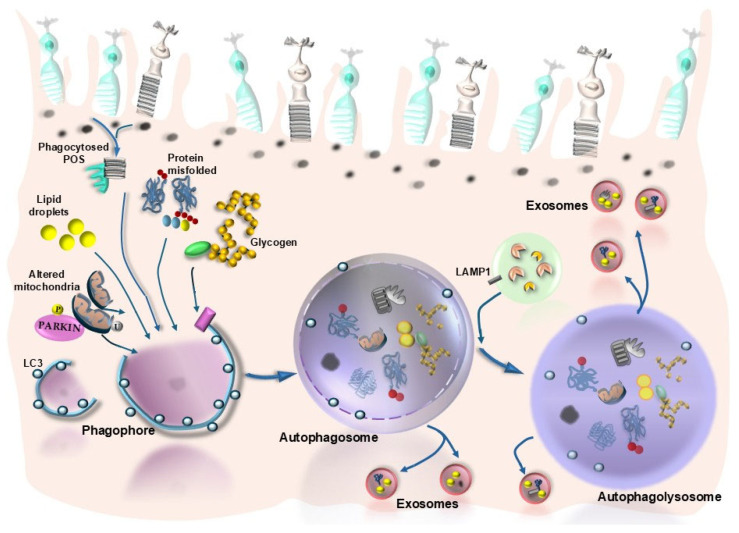
Scheme of RPE autophagy reporting sites where autophagy dysfunction triggers cell pathology in AMD. The quite generic pattern of physiological retinal autophagy progression consists of the shuttling of the lipid membrane of the photoreceptor outer segment (POS), along with other lipid content within lipid droplets, entering the nascent autophagosome (phagophore), which is stained for the presence of microtubule-associated light chain protein 3 (LC3). Within autophagosomes lipids are stored along with misfolded proteins, glycogen metabolic by-products, altered mitochondria and other constituents mentioned in the text. The relevant entry of lipid droplets and POS may occur directly into lysosomes, which otherwise merge with autophagosomes. The clearance of these constituents in the lysosome may be defective in the course of AMD. This generates abundant lipid droplets and glycogen granules along with culprit proteins within RPE cells. Again, this defective clearance leads to activate a non-canonical autophagy step, which leads to autophagy-dependent exocytosis, where lysosomes, multivesicular bodies (MVBs) or other membrane limited organelles are extruded either towards the Bruch’s membrane (likely generating drusen) or towards the opposite domain, which intermingles with the outer segment of photoreceptors (likely generating pseudodrusen).

As mentioned, roughly half of the manuscripts dealing with autophagy and retinal degenerative disorders focus on AMD, providing a significant drive to dedicate the present manuscript to discuss the role of autophagy in sustaining retinal integrity and counteracting AMD. When analyzing autophagy in the retina, one is suddenly impressed by its vast implications in several steps involved in cell metabolism and whole-cell remodeling (plasticity). Therefore, a preliminary list of various effects of autophagy, which are crucial in promoting retinal integrity or counteracting retinal degeneration, is reported here (see also Figure 1): (i) Retinal autophagy is implicated early in the development of the retina [5]. (ii) Autophagy sustains retinal anatomy [17]. (iii) Autophagy promotes retinal protein clearance [17,26]. (iv) Autophagy enhances the metabolism of retinal lipids [27,28]. (v) Retinal autophagy modulates sugar biochemistry [11,12,13,29,30]. (vi) Retinal autophagy is critical in the turnover of a number of organelles, with an emphasis on mitochondria [31,32,33]. (vii) Autophagy promotes the removal of oxidized by-products from retinal cells [11,12,13,34,35]. (viii) In the retina, autophagy is related to exocytosis, in the process defined as autophagy-dependent exocytosis [36,37,38]. (ix) Retinal autophagy regulates phototransduction and visual acuity [39,40]. (x) Autophagy modulates synaptic transmission between retinal cells [41,42]. (xi) Autophagy modulates retinal neovascularization [43]. The vast implications of autophagy at the level of multiple retinal targets explain why a defective autophagy may represent a final common pathway to produce cell pathology within retinal cells (Figure 1 and Figure 2). This calls for a systematic analysis to detail which and where specific alterations are produced in the retina due to autophagy disruption. Within this context, it is relevant to assess whether these alterations correspond to retinal alterations in the course of AMD. This point will be discussed in the following paragraphs, where specific types of autophagy, which may be implicated in the course of AMD, will be analyzed. In the second part of the manuscript, an attempt will be made to interconnect these alterations and assess whether specific kinds of cell pathology in the course of AMD may be the direct consequence of a dysfunction in retinal autophagy. In this context, an emphasis will be placed on the role of autophagy dysfunction in producing a defect in visual acuity. Autophagy alterations within cells forming the retinal pigment epithelium (RPE) will be specifically analyzed as a disease culprit at the onset of AMD [44] (Figure 3). In fact, the RPE is the first retinal layer, which appears to be involved in AMD. This is evident by the thinning of RPE, which features atrophic cells and a loss of RPE continuity. Cell pathology occurring in RPE cells of AMD patients mainly consists of an accumulation of lipid droplets along with abundant glycogen granules, aberrant mitochondria, decreased ribosomes, misfolded protein aggregates [45], and increased stagnant autophagosomes due to an impaired autophagy flux.

**Figure 4:**
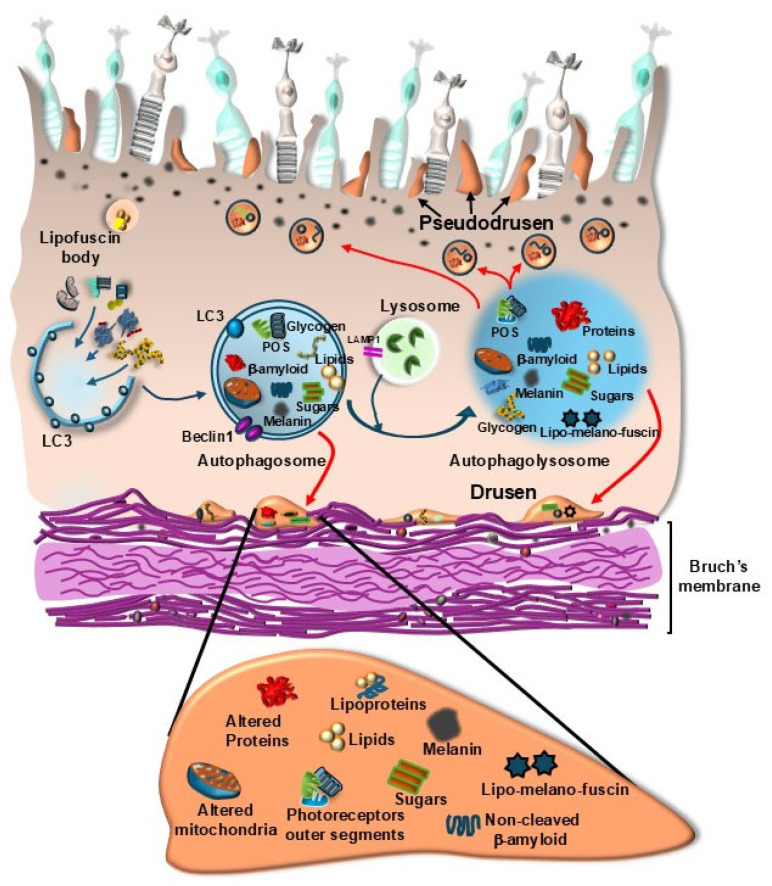
The generation of drusen and pseudodrusen in AMD. Site- and structure-specificity of drusen is closely related to RPE [48]. Drusen are produced mainly by secretory autophagy, where cell cargoes are released via the plasma membrane [47]. Drusen accumulate within the thin space underlying the basal membrane of RPE, just above the Bruch’s membrane, or alternatively, the space between RPE and the outer segment of the photoreceptors (pseudodrusen). The structure of drusen and pseudodrusen matches the content of all lysosomes and MVBs.

This is further witnessed by a loss of ability to decrease p62 levels during starvation [45]. In AMD, late autophagy vesicles are enlarged and intensely stained for lysosome-associated membrane protein 1 (LAMP-1) [46] (Figure 3). These stagnant lysosomes and multivesicular bodies (MVBs) cannot be processed within RPE cells; therefore, they are rather secreted from the cells in the process of secretory autophagy [47] (Figure 3). The occurrence of secretory autophagy is supposed to produce site- and structure-specificity in pathological hallmarks of AMD called drusen (Figure 4). These consist of extracellular amorphous aggregates, which are closely related to RPE [48]. Thus, secretory autophagy may be defined as a non-canonical autophagy pathway where cell cargoes are released via the plasma membrane rather than being degraded by lysosomes [47]. This explains why the specific site of drusen formation corresponds to the thin space underlying the basal membrane of RPE, just above the Bruch’s membrane, or alternatively, the space between RPE and the outer segment of the photoreceptors (pseudodrusen) (Figure 4).

**Figure 5:**
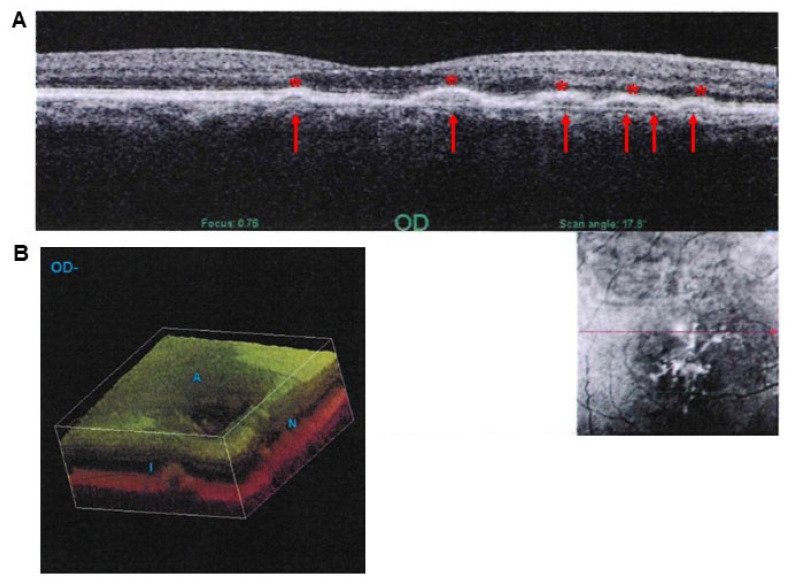
Imaging of the right retina as a representative image of AMD. (**A**) The occurrence of drusen, below the RPE between retina and choroid, is visualized in a representative optical coherence tomography (OCT) from a 68-year-old woman, who gave her informed consent. In addition, from the same patient, the retinal cube is presented (**B**). This corresponds to a computer-aided manipulation of the macular cube, which is defined by the volume of the macular region that equals 1 mm^3^. In the macular cube, it is possible to count drusen based on a single plane obtained by OCT, which transforms the region of interest from a volume into an area. The retinal pigment epithelium is evidenced in red by computerized processing in the macular cube. The occurrence of drusen in the OCT is expressed by red arrows. At this level, it is also evident that the thinning of the RPE and the decreased length of the photoreceptor layer (indicated by red asterisks) above the drusen. The patient suffers from a loss of visual acuity (best corrected visual acuity, BCVA = 20/50) and she perceives spread longitudinal temporal metamorphopsia.

Thus, extracellular aggregates of amorphous material are produced below the RPE, at the border between the outer retina and inner choroid. In addition, similar deposits may be found above the RPE interspersed within the photoreceptor outer segment (POS). The latter represent the so-called pseudodrusen or drusenoid aggregates [49]. When performing retinal optical coherence tomography (OCT) in a person affected by AMD, classic drusen appear as round, quite geometric accumulations under the RPE, while pseudodrusen (geographical sub-retinal drusenoid deposits) are interconnected accumulations, which are placed above the RPE, at the level of the distal segment of rods and cones [16,50]. In these patients, the thickness of drusen may be demonstrated through various image manipulations (Figure 5).

Concerning the structure, drusen and pseudodrusen match impressively all biochemical substrates expected following autophagy failure, which derive from inefficient lysosomal clearance [51] (Figure 4). In detail, drusen are composed of altered cellular proteins, lipoprotein particles, lipids, and sugars, and they contain photopigments, lipofuscins, and neuromelanin (lipomelanin aggregates) in addition to altered mitochondria and various vesicle remnants [52] (Figure 4). Therefore, both site-specificity and biochemical structure of drusen witness for an intracellular biogenesis at the level of RPE cells to engage the border of outer retina (pseudodrusen)/inner choroid (drusen). A seminal contribution confirming the pivotal role of autophagy in causing AMD was published a few weeks ago, while writing the present manuscript, by Bammidi et al. [53], who suppressed autophagy selectively within RPE cells. In detail, based on the hegemonic suppression of multiple autophagy activities by the protein complex named mechanistic target of rapamycin (mTOR) isoform complex 1 and complex 2 (mTORC1 and mTORC2), these authors produced a strain of mice, which was genetically manipulated to overexpress both mTORC1 and mTORC2, specifically within RPE cells. Since mTORC1 and mTORC2 are powerful autophagy inhibitors, the overexpression of both isoforms of mTOR produces a powerful autophagy suppression, selectively, within RPE cells. This mimics all the typical hallmarks of AMD, including sub-retinal deposits and lysosome dysfunction with cell death of contiguous photoreceptors [53]. When inhibiting the overwhelming mTOR activity within these RPE cells by administering torin1 to these mice, RPE cell pathology is counteracted, and AMD is no longer mimicked [53]. This manuscript represents the state of the art concerning the proof of concept about the relevance of defective autophagy within RPE as a pivot to induce AMD. The dissection of various autophagy-driven activities in producing a deleterious synergism to cause the neurobiology of AMD is reviewed in the following paragraphs.

## 3. Protein Misfolding/Unfolding, Autophagy, and Retinal Degeneration

**Figure 6:**
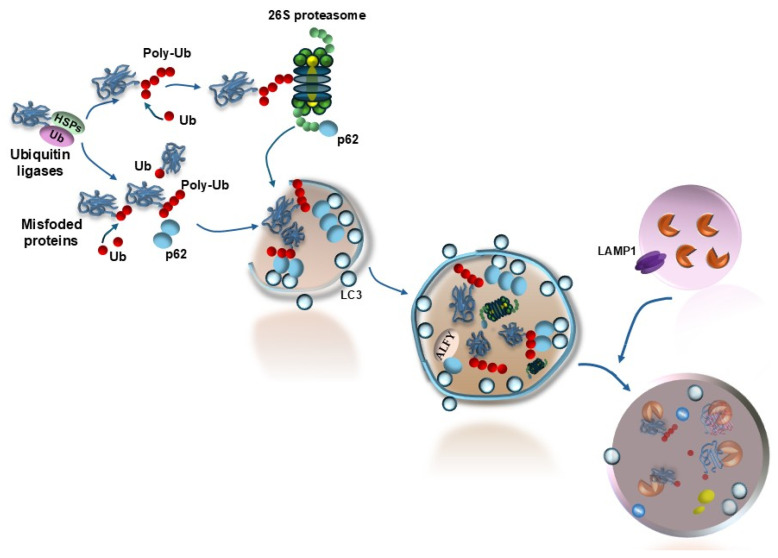
The role of retinal autophagy in removing altered proteins. The natural progression of protein autophagy consists of the binding of an altered/misfolded protein structure with ubiquitin protein or a chain of poly-ubiquitin in the process named protein ubiquitination [54]. The latter process received specific attention recently, since protein ubiquitination is required to address protein clearance both via the proteasome and autophagy pathways. In detail, the recruitment of altered proteins by the proteasome is based on the poly-ubiquitination, while poly/mono-ubiquitination addresses altered proteins to the autophagy vacuoles. As recently shown, and described in further detail in the main text, the proteasome itself, bound to a poly-ubiquitin chain and a misfolded protein, is fully shuttled within the autophagosome. This occurs massively following autophagy activation (via mTOR inhibition) to form a merged clearing organelle named autophagoproteasome through a shuttle protein named p62 (sequestosome), which binds the vesicle membrane, mainly through its Uba domain. As a result, an empowerment of enzyme activity is obtained by adding proteasome proteolytic enzymes to the enzymes occurring within the lysosomal compartment, indicated by the lysosome-associated membrane protein 1 (LAMP1).

It is now evident that altered protein homeostasis in the retina leads to degeneration [54]. This is evidenced by the altered removal of misfolded/unfolded proteins due to a primary defect in protein structure, protein folding, and protein ubiquitination [54]. The latter process received specific attention recently, since protein ubiquitination is required to address protein clearance both via the proteasome and autophagy pathways (Figure 6).

A defect in protein ubiquitination results in several concomitant events, which produce an excess of protein accumulation. When a protein substrate undergoes a covalent binding with ubiquitin, a chain of chemical reactions takes place, and several fundamental processes in the cell are altered, such as gene transcription, DNA integrity, cell proliferation, cell signaling, and cell death. A defective autophagy impedes these processes, and it is strongly bound to impaired protein clearance. As recently demonstrated by Feng and colleagues [55], when stressful conditions in the cell occur, several autophagy components aggregate along with altered substrate proteins (stress granules) to initiate the autophagy-dependent degradation of altered substrates. This process represents a primordial defense mechanism, which occurs in plants as well as within retinal mammalian cells [55,56]. For instance, mutations in the CERKL gene, which are responsible for RP, produce a defect in protein aggregation within stress granules [57] and a failure of autophagy [58], which leads to altered protein removal [59]. It is remarkable that, even in the case of RP, the critical site for autophagy dysfunction is the RPE, where the mutant CERKL gene sorts most of its deleterious effects [59]. Mounting evidence indicates defective protein autophagy, which is constantly present in AMD [9,11,12,26]. In detail, altered autophagy appears to be critical mostly when occurring within RPE cells [9,12,16,26,59,60]. The significance of altered autophagy as a cause of impaired protein degradation is expected to lead to abnormal protein aggregation, loss of protein function, and abnormal protein spreading, thereby affecting visual processing in the retina. A defect in the process of autophagy initiation leads to an impairment of the shuttling of various proteins via binding with p62 (Figure 6), which recognizes the autophagosome for protein clearance [61]. In physiological conditions, even the proteasome structure and proteasome activity enter the autophagosome following autophagy initiation to provide complete protein clearance [15,61,62,63,64]. The downstream organelle named autophagoproteasome possesses enriched proteolysis provided by both proteasome and autophagy pathways [65,66] (Figure 6). Abnormal amounts of various proteins, including α-synuclein, within exosomes are demonstrated in the pathology of the aged retina, where they accumulate with *tau* within ubiquitin-positive inclusions, as shown by Leger et al. [67], in the course of autophagy inhibition. Vice versa, in physiological conditions, α-synuclein deposits in the cell are promptly metabolized due to quick autophagy activation [65]. When autophagy activation is enhanced by administering the mTOR inhibitor rapamycin, a massive merging occurs between misfolded proteins, autophagy, and proteasome components as evident by co-immunoprecipitation of misfolded α-synuclein with p62, proteasome, and LC3 and an impressive merging of immunofluorescence for proteasome and LC3 vacuoles [65]. Conversely, autophagy and proteasome components fail to merge and dissipate following autophagy inhibition or retinal damage induced by the autophagy-inhibiting neurotoxin 1-methyl, 4-phenyl, 1,2,3,6-tetrahydropyridine (MPTP) [65,68]. Similarly, impaired protein clearance was early emphasized in relation to the accumulation of the protein β-amyloid [69] and non-fibrillary amyloid oligomers [70], which eventually are secreted from RPE to be a part of drusen in AMD. Thus, the impact of autophagy impairment concerning protein clearance is likely to underlie the exocytosis of protein-engulfed autophagolysosomes to produce extracellular drusen, where specific proteins, including non-cleaved β-amyloid, are aggregated [15]. The autophagy-related structure of drusen is revealed directly by immunostaining for proteasome/autophagy-related proteins, such as ubiquitin [71], ATG5 [72], poly-ubiquitin, and p62 [73]. In line with this, Ren et al. [74] classified AMD as a proteopathy, where the mutual role of proteasome and autophagy in removing altered proteins is suppressed [62,71]. This is in line with the novel scenario of an mTOR-dependent metabolic unit condensing autophagy and proteasome activity within specific cell domains, the autophagoproteasomes [61,62,63]. In addition to a defect in protein clearance, oxidized lipoproteins per se may induce a decrease in protein synthesis within RPE cells, thereby worsening the course of AMD [45]. This is in line with the pathological signature of retinal degeneration, which is mostly based on the immunodetection of specific protein antigens due to a loss of protein clearance. Abnormal protein metabolism may be further related to a dysregulation of protein degradation within the extracellular matrix (ECM), which increases the deposition of the abnormal material forming drusen over time [11,12,13,75]. In these conditions, abnormal proteins are secreted rather than being metabolized by RPE cells, in the course of AMD. Among these, the ECM protein collagen XVIII is very important, considering how autophagy activity provides integrity of the RPE. This is indicated by the findings of Kivinen et al. [71], who showed that in collagen XVIII knock-out mice, age-related RPE degeneration with the accumulation of drusen-like deposits occurs. As reported by Kaarniranta et al. [15,76] in the chronic course of AMD, the autophagy process is immensely important for RPE cell survival, also concerning autophagolysosomal-mediated clearance. The occurrence of abnormal protein deposits in AMD, despite being important due to a high number of different proteins, which can be stained as hallmarks, is likely to be over-emphasized since the total amount of proteins within pathological inclusions (drusen) is limited when compared to other chemical species, such as lipids. The intense research on the nature of proteins within drusen is rather related to specific immunostaining and to establishing a chain of enzyme-driven biochemical events to dissect the functional progression of the neurobiology of AMD. The amount of abnormal extracellular proteins may be toned down when considering that lipids remain the larger constituent of drusen. Thus, the present manuscript is called to address such a point.

## 4. Lipids Autophagy (Lipophagy) and Retinal Degeneration

**Figure 7:**
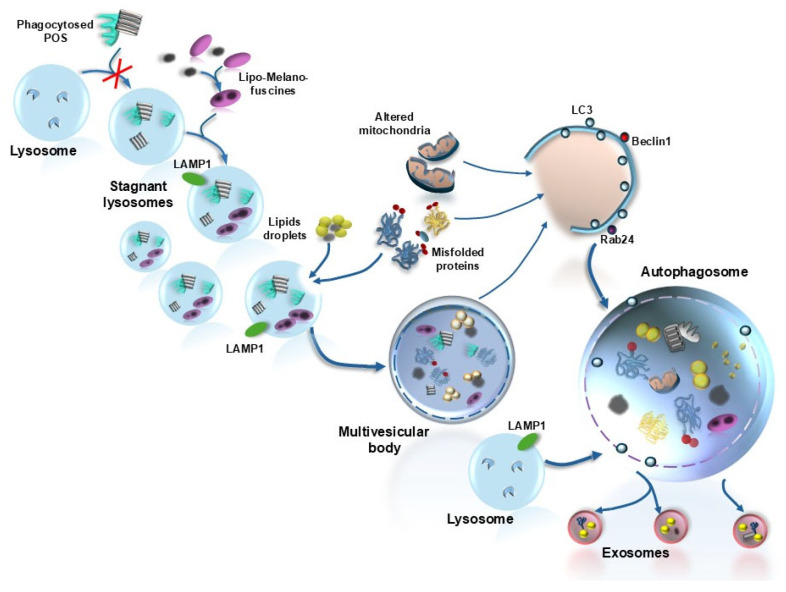
Specificity of retinal lipid autophagy (lipophagy). In the retina, lipophagy consists of a specific activity of the autophagy machinery leading to degradation of the photoreceptor outer segments (POS), which contribute largely to forming lipid droplets within RPE cells. The conspicuous amount of lipid droplets is taken up by the lysosomal compartment upon the merging of lysosomal vacuoles and lipid vacuoles/droplets. Very often, the lipid droplets directly enter the lysosomes and MVBs, although the specific receptors remain questionable. Some recent evidence suggests that the protein family forming the vacuolar protein sorting required for transfer (VPS), such as VPS4A found in the liver, may be strongly involved, along with the early autophagy inducer beclin1. One might consider that in baseline conditions, retinal autophagy, while granting the clearance of some misfolded proteins, is strongly engaged in taking up and degrading innumerable lipid droplets. When autophagy is impeded some protein aggregates persist although the lipids engulf the cell. This explains why the secretory autophagy of lipids leads to the formation of drusen, where lipids represent almost half of the whole volume.

As recently defined [77], lipophagy consists of a specific activity of the autophagy machinery leading to the degradation of lipid droplets by the lysosomes compartment upon the merging between lysosomal vacuoles and lipid vacuoles/droplets (Figure 7).

**Figure 8:**
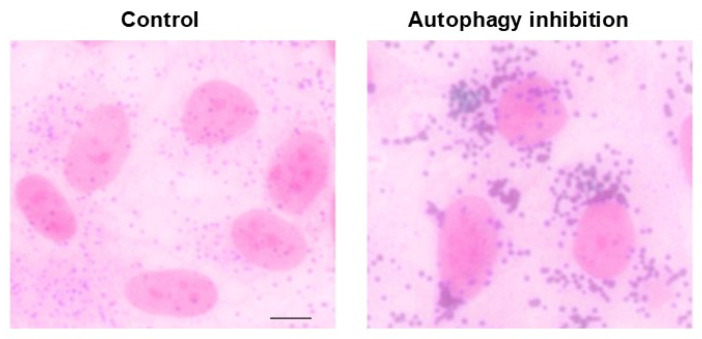
Representative staining of lipid droplets following autophagy inhibition (3-methyladenine, 3-MA, 10 mM) in human RPE cell line (ARPE). The staining of lipids was obtained with Sudan Black B (8 min exposure), while nuclei were counterstained with a Nissl-type approach (Fast Red, 7 min). Scale Bar = 15 μm. Original picture by M.F. and G.L.

The receptors, which bind lipid droplets within lysosomes, remain, at present, a matter of debate [77]. Recent evidence suggests that a vacuolar protein sorting 4 homolog A (VPS4A) may represent the lipophagy receptor on autophagosomes, at least within liver cells [78]. In line with this, VPS4 within the mammalian retina was reported to regulate the transport within MVBs [79], although its activity on retinal lipid droplets remains to be established. The occurrence of high amounts of lipid droplets is typical within RPE cells in the course of AMD [47], and they are replicated following autophagy inhibition (Figure 8).

**Figure 9:**
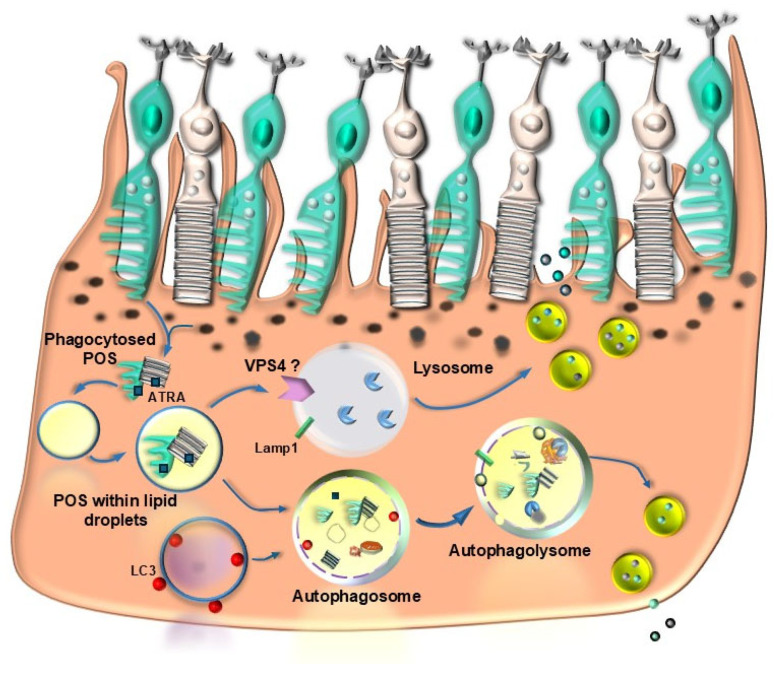
Lipophagy within RPE cells. Lipid droplets are well evident within RPE cells in baseline conditions and are in excess during AMD. Lipid autophagy is fundamental in maintaining the viability of RPE cells since lipids are abundant in the physiology of RPE. Several systemic sources, such as the bloodstream and lymphatic stream in the choroid of patients affected by dyslipidemia, deliver lipids to the RPE. However, the greatest amount of lipids is specifically driven by an excess of lipid substrates entering the RPE from the outer segment of photoreceptors (POS). A major constituent of photosensitive disks is retinoic acid in the form of its aldehyde. Remarkably, all-trans aldehyde derived from retinoic acid (all-trans-retinoic acid, ATRA) stimulates autophagy. These lipids may enter either the autophagosome or the lysosomal compartment. Indeed, when lipids are in excess and coalesce to form lipid droplets, the preferential pathway consists of the direct entry into lysosomes, where the VPS-related receptor is the candidate receptor for lipid droplets. The storage of lipid-rich lysosomes, lipid-rich VPS, and frankly abundant interspersed lipid droplets within RPE cells is likely to trigger non-conventional secretion and non-canonical secretory autophagy to build up at least half of the whole drusen volume.

In line with this, Mitchell et al. [80] previously reported a dysregulation of lipid metabolism as a major risk factor for developing AMD, while high amounts of dietary lipids are known to be associated with increased prevalence of AMD [81]. In this latter study, the deleterious effects of an excess of lipids within RPE cells appear to be mostly relevant for mitochondrial activity. Several lipids are involved in mitochondrial alterations associated with AMD. This is evident when considering drusen composition. The amount of lipids within drusen surpasses 40% of the whole drusen volume [82], which is in excess compared with total proteins, sugars, organelles, and neuromelanin [15]. In keeping with this, a main homeostatic function of RPE cells is manifest by lipophagy, which consists of phagocytosing lipid-rich end-products clustered within lipid droplets (Figure 7). These may potentially derive from several sources, such as the bloodstream in the choroid of patients affected by dyslipidemia and the physiological excess of lipid substrates usually entering the RPE from the outer retina. In baseline conditions, though, most lipids entering the RPE derive from lipid membranes forming the disks or invaginations within outer segments of rods and cones, respectively (Figure 9).

**Figure 10:**
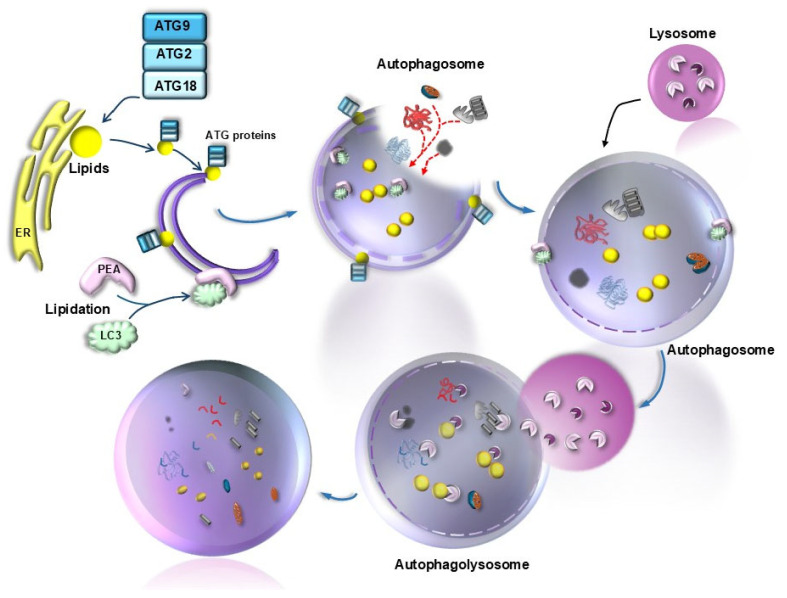
Dual effects of lipids on retinal autophagy. While lipids are substantial substrates for the autophagy within RPE cells, which mostly recruits a direct lysosome clearance, the activity of some phospholipids, such as phophatidylethanolamine (PEA), is a powerful inducer of LAP autophagy. PEA generates the lipidation of LC3, which feeds the nascent phagophore membrane and modulates its membrane bending, leading to ceiled autophagosomes.

The recycling of the actual platform for phototransduction strongly involves the role of lipid autophagy within RPE cells. It is postulated that such a turnover participating in the visual cycle is associated with MAP1LC3 (microtubule-associated protein 1 light chain 3)-associated phagocytosis (LAP; Figure 9) [83,84]. In line with this hypothesis, decreased LAP was suggested to be crucial in triggering RPE degeneration during AMD [80,85]. On one side, the occurrence of a great amount of lipids within drusen is the consequence of altered lipophagy; on the other side, lipids are detrimental to the efficacy of autophagy itself, thus producing a vicious cycle. Triglycerides (TGs), which aggregate following autophagy inhibition, further inhibit the autophagy flux [86]. Phototransduction, which occurs at the level of the outer segment of photoreceptors, converts retinoic acid from the 11-cis-aldehyde isomer into the all-trans-aldehyde (all-trans-retinaldehyde). Remarkably, all-trans aldehyde derived from retinoic acid (all-trans-retinoic acid, ATRA) stimulates autophagy. A study by Tzankov [87] found a disruption of Golgi apparatus vacuolization following retrograde transport of ATRA. It would be interesting to probe the co-localization of ATRA with the lipophagy inducer VPS4A. This suggests that the lipid-rich photoreceptor outer segment of rods and cones, rich in ATRA, when phagocytosed by RPE, directly stimulates autophagy (lipophagy) within RPE cells (Figure 9). A seminal work by Tokarz et al. [88] indicates that retinoic acid within the photoreceptor outer segment is related to autophagy within RPE. In line with this, a recent study [89] indicates that retinoic acid acts as a powerful autophagy inducer, which promotes epithelial differentiation. These findings are relevant when moved to the retinal level, where autophagy stimulation blocks the transition from RPE cells to mesenchymal cells, which is seminal for maintaining retinal integrity. Thus, maintenance of RPE integrity likely depends on the amount of lipophagy under the influence of photopigment turnover. Conversely, when lipophagy is inhibited, one would expect that RPE to undergo a mesenchymal transition (MEPT), which fosters RPE degeneration as occurring in diabetes due to high glucose levels [90]. It is remarkable that, apart from baseline conditions, the levels of MEPT are crucial in AMD since elevated MEPT is a hallmark of RPE cells during AMD. The occurrence of MEPT is critical in shifting the phenotype of AMD from the dry toward the wet isoform, which is characterized by neo-angiogenesis [26,91,92]. In detail, when autophagy is inhibited, a dismantling of desmosomes and hemi-desmosomes of RPE cells occurs, which are released via exosomes upon lysosomal engulfment, forming the basis for the occurrence of MEPT [92]. Such an autophagy-dependent process is regulated via mTORC1/2 [26]. The enormous relevance of lipid metabolism for retinal disorders was deeply analyzed by Gabrielle [93]. In the present context, we wish to emphasize the relevance of lipophagy in AMD, for this topic has a dual relevance, since lipids are both substrates and modulators of autophagy (Figure 10).

**Figure 11:**
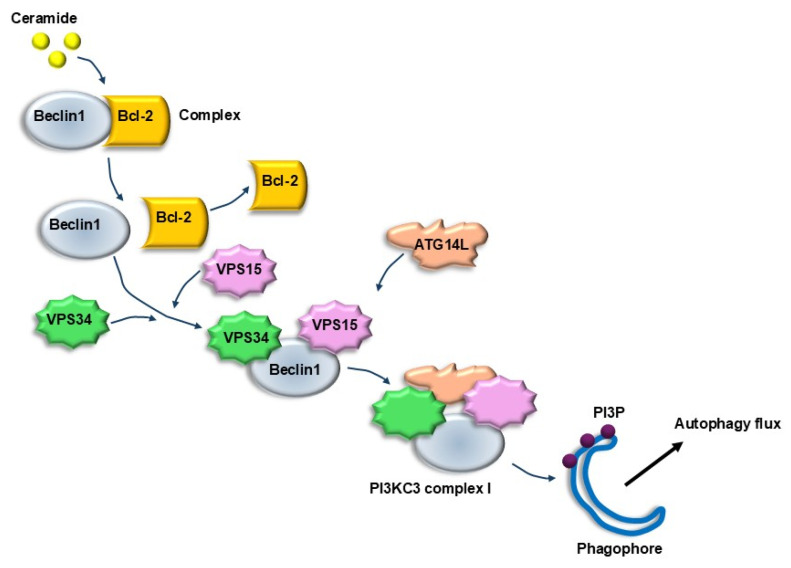
The sphingolipid ceramide stimulates autophagy by acting on Beclin1. Ceramide promotes the removal of Beclin1 from the Beclin1/Bcl-2 complex. This finally leads to the formation of a multi-protein complex (known as Beclin1-VPS34-VPS15), which stimulates autophagy [102].

In fact, during early stages, the autophagosome is activated by a lipidation process, which is produced by the binding of phosphatidylethanolamine (PEA) to generate lipidated LC3 (LC3II), which enlarges the nascent autophagy vesicle, while fostering the bending of its membrane (Figure 10). The multiple roles of lipids in various steps of autophagy initiation and progression were elegantly reviewed by Jarocky et al. [94]. The relevance of lipids as autophagy substrates, despite being recently intensely investigated, was early suggested by the fact thatbeclin1 is placed in close relationship with lipid droplets. These concepts provide a general glimpse of the essential role of lipophagy and explain the abundance of non-metabolized lipid droplets due to autophagy failure in AMD (Figure 8). In fact, in patients affected by AMD, RPE cells feature an excess of lipid droplets (due to a defect in lipid clearance) along with giant autophagosomes (due to a defect in autophagy progression) [47]. Lipid droplets, which are the substrates of lipophagy [95,96], are increased within AMD-RPE cells along with large cell vacuoles [47]. These confluent droplets provide AMD-RPE cells with wide cell areas, where a decrease in cytosol density is observed. These very same findings concerning lipid droplets are observed within RPE cells following autophagy inhibition (Figure 8), while a direct demonstration at electron microscopy in the ARPE cell line is a matter for future experiments. The involvement of altered lipophagy and altered lipid metabolism in AMD is supported by the highest prevalence of AMD in the course of the metabolic syndrome, where lipid metabolism is altered with an increase in the plasma levels of triglycerides and cholesterol, which are known to inhibit autophagy [22,97]. Conversely, the role of Apolipoprotein-E (APO-E) in AMD is beneficial due to its activity in removing lipids from RPE [35]. APO-E deficiency is bound to the development of AMD, and in keeping with this, mice knocked out for the APO-E gene develop experimental AMD, which can be reversed by autophagy tethering compounds [35,98,99]. The relevance of APO-E for lipophagy in AMD is further confirmed by the high amount of APO-E operating within RPE cells in baseline conditions to shuttle lipids across cell membranes, thereby exerting a protective effect [98,99]. Several lipids are involved in modulating retinal autophagy. This is the case of phosphoinositides [100], sphingolipids [94], oxysterols [101], and triglycerides [27,86], which all feature altered metabolism in aging, likely contributing to the onset of AMD [94]. A significant, quite specific effect on autophagy is promoted by some sphingolipids, such as ceramide, as reported by Jarocky et al. [94]; ceramide promotes the removal of Beclin1 from the Beclin1/Bcl-2 complex. This allows Beclin1 to interact with VPS34 and VPS15, leading to the formation of a multi-protein complex (known as Beclin1-VPS34-VPS15 complex) (Figure 11), which is a powerful autophagy stimulator [102].

As suggested by Jarocky et al. [94], this may explain why the estrogen antagonist tamoxifen, which increases the endogenous availability of ceramide, promotes autophagy. Again, confirming the pivotal role of beclin1 in lipid metabolism, and VPS in binding lipid droplets to autophagosomes, the interaction of Beclin1 with VPS is crucial in inducing lipophagy. This is in line with the seminal role of the VPS-interacting protein Beclin1 in AMD. The activity of Beclin1 in AMD is crucial since it leads to autophagy stimulation and counteracts disease progression. Relevant proteins to induce lipophagy are reported in Table 1.

Apart from lipids, such an effect is partly grounded on the interaction of Beclin1 with AMBRA1 to promote mitochondrial autophagy (mitophagy) [31]. Despite the promising role of Beclin1 in sustaining retinal integrity and counteracting AMD, it is a pity that no study so far has investigated whether genetic depletion of Beclin1 may mimic AMD in aged mammals. In addition to an increase in lipid droplets, RPE cells affected by AMD often feature the presence of an excess of glycogen granules, which occur in high amounts in RPE cells in baseline conditions and massively augment when autophagy is occluded. This is expected due to a strong energy demand within RPE cells. Therefore, the failure of sugar clearance related to an autophagy defect will be analyzed in the next paragraph.

## 5. Glycation End-Products, Autophagy, and Retinal Degeneration

**Figure 12:**
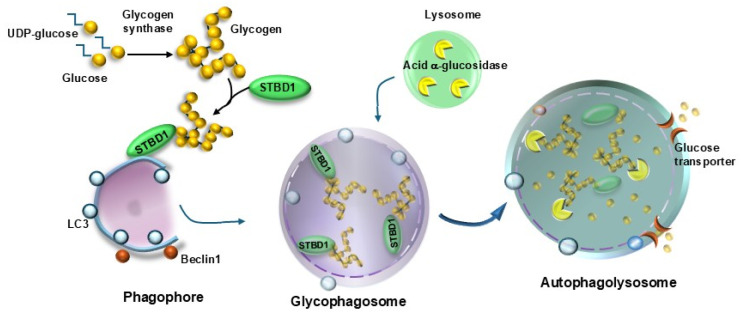
Glycogen-specific autophagy (glycophagy). Glycogen chains in the cell are built up by adding moieties of uridine diphosphate (UDP)-glucose as a substrate. These primary chains of glucose become very long through the progressive addition of subunits through the enzymatic activity of glycogen synthase. The main chain is further branched in its secondary chains by the activity of glycogen-branching enzyme. The autophagy degradation of these glycogen long chains occurs via recognition with a specific receptor for glycogen, named starch binding domain-containing protein 1 (STBD1). This binding addresses cytosolic glycogen towards the autophagy machinery (glicophagy), once it is stored within LC3 and beclin1-positive autophagosomes. In this way, a specific phagosome is formed, which is named glycophagosome. Following autophagosome–lysosome fusion, a specific enzyme named α-glucosidase mediates lysosomal glycogen breakdown [112,113]. Single glucose molecules may enter directly into the lysosome through a specific glucose-sensing site on the lysosome.

The occurrence of abnormal proteins within cells and the extracellular space goes beyond protein conformation to recruit protein glycation due to abnormal sugar metabolism. The formation of glycation end-products (AGEs) is described in AMD [103]. This is relevant and poses glycation stress as a major determinant of AMD [103,104]. In fact, the process of glycation recruits various chemical species, and it occurs due to spontaneous non-enzymatic binding between proteins, lipids, nucleic acids, and abundant sugars or sugar derivatives [105,106,107,108]. This is due to an autophagy defect altering the biochemistry of carbohydrates, as also witnessed by the glycation of altered proteins within RPE cells in the course of AMD [109] and the constant presence of carbohydrates and AGEs within drusen structures [15]. Due to the high metabolic demand produced by the great energy needs within RPE cells [104,110,111], sugars are abundantly present, and their metabolism, coupled with mitochondrial activity, is critical for promoting the integrity of RPE cells. Before accessing mitochondria, the metabolism of sugars is mainly operated through the autophagy control of the glycolytic pathway. Sugar-induced autophagy activation within RPE cells occurs via a glucose-sensing activity placed on lysosomes (Figure 12) [112,113].

When autophagy is directed toward sugar metabolism (glycophagy), this occurs through the activation of the glycogen-sensing activity of autophagosomes and the glucose-sensing of lysosomes (Figure 12), which become committed to degrade complex carbohydrates (Figure 12). When compared to the clearing activity of lysosomes toward proteins, which is well characterized, only a few and very recent reports have analyzed the lysosome clearance of sugars [113,114,115,116]. Nonetheless, this novel scenario is in line with multiple binding sites between autophagosome, lysosomal membrane and long-chain carbohydrates such as glycogen, which are compatible with the bending of the lysosomal structure around glycogen granules, which may be described by analyzing the ultrastructure of autophagy-deficient RPE cells. Apart from AGEs formed de novo within RPE cells, this retinal layer can take up circulating AGEs through specific receptors (RAGEs). This mechanism is critical to regulating the amount of AGEs that enter the RPE cells from the choroidal bloodstream. In fact, in clinical studies, the onset of AMD is also related to either the presence of high amounts of glucose in the bloodstream and/or the specific polymorphisms of RAGE [117]. This is supposed to have a relevant effect in the course of the metabolic syndrome, where abnormal glucose tolerance exists along with dyslipidemia, and a higher risk for AMD is described [118]. Similarly, in the course of diabetes, the occurrence of a specific diabetic retinopathy is concomitant with a higher risk of developing AMD [119]. The deleterious effects of AGEs extend outside the RPE cells since a specific accumulation of AGEs is described at the level of the Bruch’s membrane, where it strongly contributes to the thickening of Bruch’s membrane [120], which represents another hallmark of AMD along with atrophy of the RPE and photoreceptor cell loss.

## 6. Mitochondria, Autophagy, and Retinal Degeneration

Among the pathological hallmarks of AMD, altered mitochondria within RPE are constantly described. The loss of functional mitochondria is the kernel for the bioenergetic crisis affecting RPE cells in AMD [121]. This is directly witnessed by the analysis of RPE cells in primary cultures from AMD donors compared with controls. AMD-RPE cells possess a severe disintegration of mitochondria [46], which accompanies the accumulation of lipid droplets and glycogen granules described above. In detail, a dramatic increase in the number of damaged mitochondria is present within RPE cells from AMD patients [46,122], which can be reproduced within the RPE cell line following autophagy inhibition, as reported in the representative Table 2.

Cultured cells from the human normal Adult Retinal Pigment Epithelial cell line-19 (ARPE-19, Biobanking of Veterinary Resources, Brescia) were analyzed following treatment either with 10 mM 3-methyladenine (3-MA) or vehicle (Control) for 72 h. At the end of the treatments, cells were fixed with 2% paraformaldehyde and 0.1% glutaraldehyde for 90 min and centrifuged to obtain cell pellets, which were post-fixed in 1% OsO_4_ for 1 h at 4 °C. Then, cells were dehydrated in ethanol solutions and embedded in epoxy resin. Ultrathin sections (90 nm thick) were collected on nichel grids, stained with uranyl acetate and lead citrate and then examined using a JEOL JEM SX100 transmission electron microscope (JEOL, Tokyo, Japan).

The count of total, healthy and altered mitochondria was carried out at 6000× magnification by scanning each examined grid starting from a corner in parallel sweeps to examine at least 30 cells per group. Briefly, mitochondria were identified by TEM for the presence of a typical double-membrane limiting an inter-membrane space and an area internal to the inner membrane, where the matrix is interrupted by crests, in a sort of labyrinth.

Data were given as the mean ± SEM per cell counted in 30 cells for each experimental group. Comparisons among different groups were carried out with one-way analysis of variance (ANOVA), followed by Scheffè’s post hoc analysis. The null hypothesis (H_0_) was rejected for *p* < 0.05.

It is remarkable that, in AMD, as well as in RPE cells undergoing autophagy inhibition, mitochondrial alterations are accompanied by a net decrease in total mitochondria. This is intriguing since one may expect that autophagy (mitophagy) failure may lead to the accumulation of non-digested mitochondria due to altered removal. It is well known that mitochondrial removal is tightly related to the biogenesis of novel mitochondria. The mitochondrial biogenesis is strongly dependent on autophagy activation. The pathways that regulate mitochondrial removal through (PINK1/Parkin interaction) and mitochondria biogenesis through peroxisome proliferator-activated receptor gamma coactivator 1-α (PGC1-α) activation are interconnected. Although within RPE cells, autophagy inhibition appears to result in a moderate suppression of the removal of altered mitochondria, such a moderate suppression of mitophagy is surpassed by a strong inhibition of mitochondrial biogenesis. This is likely to depend on the pivotal role of AMBRA1 in regulating mitochondrial turnover within RPE cells [31]. In fact, as elegantly indicated by these Authors, AMBRA1 may promote mitophagy, either by regulating the PINK1/parkin pathway or following a PINK1-independent pathway [31]. This concept is confirmed by the finding that in AMBRA1 heterozygous mice, mitochondrial biogenesis does not occur, and the net amount of mitochondrial mass is represented by altered mitochondria [123]. This explains why in AMD patients, the levels of AMBRA1 are increased within RPE cells while a decrease of PINK1 is detected [124]. The progressive damage likely leads to a net decrease in mitochondria, where the remaining organelles, despite being reduced, possess severe alterations. The activity of mitochondria in AMD is strongly affected, as well as the decreased tolerance to oxidative stress and the higher amount of reactive oxygen species (ROS) being produced [46]. In fact, in AMD, a reversal of ATP production is reported, which shifts from the intramitochondrial respiratory chain (in controls) toward anaerobic glycolysis (in AMD). This explains the high demand for glucose since the amount of energy provided by glucose metabolism decreases eighteenfold in anaerobic conditions. Mitochondrial damage is also detectable within mitochondrial DNA (mtDNA), which is potentially transmittable from cell to cell, spreading the involvement of RPE in the course of the geographic degeneration in AMD [125]. As expected, altered mitochondria are difficult to remove since the mitophagy clearance through lysosomes is impaired as a consequence of a disrupted autophagy flux [46]. Such a lysosomal degradation decreases with age, making it likely that this phenomenon fosters the onset of AMD [126]. Thus, a defect in mitochondrial biogenesis may be considered as inherent to a defect of the autophagy pathway, which regulates mitochondrial turnover and mitochondrial biogenesis, whereas a defect in mitochondrial removal may be considered as the direct consequence of lysosomal stasis. The autophagy-/mitophagy/-dependent mitochondrial integrity is key in counteracting AMD [31]. The mitochondrial turnover in the retina is profoundly affected by the autophagy status, which provides a selective removal of altered mitochondria. This removal is largely dependent on the expression of the protein PINK1 [127] on the mitochondrial membrane, where it recruits parkin on the mitochondrial structure to initiate the removal by adding ubiquitin molecules (often as poly-ubiquitin chains). Nonetheless, mitophagy may occur through PINK1/PARKIN-independent mechanisms as well. An altered mitophagy leads to the persistence of altered mitochondria, which typically occurs during retinal degeneration. In diabetes, this appears to rely on a defective PINK1/Parkin interaction [32], while in AMD, the age-dependent suppression in the amount of AMBRA1 prevails [31]. This is in line with the protective effects on RPE cells induced by the enhanced expression of genes involved in mitochondrial biogenesis, like PGC1-α and TFAM [128]. It is remarkable that a repression of PGC-1α, which is the main activator of mitochondriogenesis, induces an experimental model of AMD in mice exposed to a high-fat diet [128]. During a normal diet, suppression of PGC1-α alters RPE cells, featuring an excess of lipid droplets and alters retinal activity [129]. PGC1-α is a powerful orchestrator of mitochondrial turnover [130]. When suppressing the expression of PGC1-α, RPE cells undergo mitochondrial dysfunction along with EMT and a metabolic shift toward increased anaerobic glycolysis [130]. The powerful effects of mitophagy to stimulate mitochondriogenesis during autophagy activation were well established by Palikaras and Tavernakis [131], who discovered how the activation of mitophagy was balanced by the biogenesis of novel, well-structured mitochondria to provide a harmonic renewal of the mitochondrial apparatus of the cell. These findings were later confirmed [132,133,134,135,136,137] and extended to the retina [138]. These manuscripts elucidated that the imbalance between mitochondrial biogenesis and degradation may induce several pathologic conditions. The aging process impairs the harmonic activity of mitochondrial biogenesis and mitophagy by triggering biochemical alterations that foster neurodegeneration. In detail, these manuscripts demonstrate that SKN-1 transcription factor regulates genes involved in both the stimulation of mitochondrial biogenesis and the stimulation of mitophagy by enhancing DCT-1 expression in *C. elegans*. In mammalian cells, BNIP3 acts as the homolog of DCT-1 as a key mediator of mitophagy through activation of the PINK-1/Parkin pathway following its ubiquitination upon mitophagy-inducing conditions to mediate the removal of damaged mitochondria. The increased number of altered mitochondria triggers SKN-1 activation, which initiates a bipartite retrograde signaling pathway, stimulating the coordinated induction of both mitochondrial biogenesis and mitophagy genes. The imbalance of both activities may lead to an excess of mitophagy in the absence of mitochondriogenesis [138]. Contrariwise, a defective mitophagy, as shown in AMBRA1 defective mice, may lead to a loss of ganglion cells and retinal degeneration [139]. Thus, the appropriate orchestration of mitochondriogenesis and mitophagy may be key in promoting retinal survival. It is interesting to note how a defective activity of AMBRA1 has been demonstrated beyond AMD in the course of CNS degenerative disorders such as Parkinson’s disease (PD) and Alzheimer’s disease (AD), [31,140,141,142].

## 7. Intersections Between Ineffective Autophagy Clearance and Drusen Formation in AMD

As we reported a few years ago [16], it appears that in AMD all the substrates (proteins, sugars, and mostly lipids) joined with cell organelles (mostly mitochondria) and cell debris, which are cleared by autophagy in physiological conditions, are prone to being engulfed by autophagosomes and ineffective lysosomes. In these conditions, it is likely that, according to a common mechanism, the abnormal secretion of non-degraded substrates may represent the unconventional solution to get rid of these overwhelming intracellular cargoes [143]. The various chemical species and cell organelles forming these cargoes may account for the polymorphic nature of exosomes released by RPE cells in the course of AMD. In turn, this may justify the occurrence of a great amount of MVBs, which are precursors of exosomes and occur within RPE cells following autophagy inhibition. The various components being released through exosomes, in turn, may explain the polymorphic nature of drusen, which accumulate between RPE and Bruch’s membrane, as we recently reported [11]. In this way, autophagy failure may lead directly to the formation of drusen, which represents the culprit of AMD [72]. This is in line with the content of drusen, which are composed of a variety of proteins and an excess of lipids, where lipofuscin and melanosomes merge to produce lipo-melano-fuscin [144]. Within stagnant lysosomes and exosomes, these lipo-melano-fuscin are likely to merge with photo-pigments, misfolded/unfolded proteins, and glycated end products to produce the early soft lipidic structure, which composes the newly deposited drusen, later evolving toward harder extracellular deposits in the course of dry AMD [26,109]. The theory that, in the course of AMD, a defective autophagy generates drusen starting from non-digested autophagy substrates is further grounded on the evidence that the very same material occurring within drusen is present within engulfed lysosomes of RPE cells [145,146,147]. This indicates that non-digested structures move from dysfunctional RPE cells to be released into the extracellular space. These concepts spread the significance of defective autophagy in AMD from the intracellular milieu of RPE cells to the pathological material observed in the extracellular space at the level of the outer retina/inner choroid. A defective autophagy within RPE is bound to marked alterations of photoreceptors, the Bruch’s membrane, and choriocapillaris (CC). In this way, the outer retina is involved along with the inner choroid in the pathogenesis of AMD [12]. In this manuscript, a novel vista about AMD is reported where the whole disease core is represented by the retinal-choroid neurovascular unit (RNVU) centered on RPE cells. As we previously indicated [12], the concept of RNVU is composed of the outer segment of photoreceptors embraced by RPE cells, which are supplied by the inner layers of the choroid (i.e., CC) [148,149,150,151]. A defect in the autophagy status may recruit all these components of the RNVU. According to the onset of AMD as a disease affecting the RNVU, a defective autophagy is expected not to cope effectively with high amounts of toxic species formed within the outer segment of photoreceptors and RPE cells. In these conditions, lipid/protein/sugar aggregates, along with stagnant inert lysosomes, cannot be retained within RPE cells, and they are released mainly towards the Bruch’s membrane in the extracellular space following a transient intracellular accumulation. A defective autophagy within the inner choroid relents the vascular glymphatic and hematic clearance of these exosomes, which persist and mature irreversibly below the retina to generate dry AMD. The persistence of extracellular aggregates below specific sites of the retina may spread geographically the pathology to the neighboring retina. This geographic atrophy is conditioned by the lack of extracellular vascular clearance to clear the inner choroid/outer retina. The physiological efficacy of the autophagy process may start within RPE, and it may extend beyond the clearance of engulfed lysosomes throughout exosomal release. The abnormal release from RPE cells may be considered in light of previous studies by Zhang and Schekman [143] and, more specifically, by Villeneuve et al. [152]. In this manuscript, the Authors report a specific secretion of exosomes, which is independent of the conventional endoplasmic reticulum–Golgi secretory pathway and does not rely on classic GRAP proteins. This exocytosis is strongly bound to the double membrane autophagy-related vesicles and represents what we previously defined as autophagy-related exocytosis. Recently, evidence has been provided that, upon mild autophagy dysfunction, mostly expressed by lysosome accumulation, this autophagy-dependent secretory activity takes place [47]. Such a process is promoted when Akt2 levels in the RPE cells increase, which suppresses lysosome activity and provides a signal to activate non-canonical autophagy clearance via secretory autophagy. Briefly, as mentioned above, the various pathways composing the autophagy system include the compensatory bypass of the lysosomal clearance through the release of non-digested cargoes. This mechanism, which is defined as secretory autophagy, is triggered within RPE cells when the canonical autophagy pathway is not fully working in the RPE cells, and lysosomes are ineffective in degrading substrates. In these conditions, the Akt2-dependent pathway promotes the transfer of engulfed vesicles towards the plasma membrane to provide an extracellular clearance. This secretory autophagy is stimulated by a protein complex demonstrated by Ghosh et al. [47], which is composed of AKT2-SYTL1-TRIM16-SNAP23. It is supposed that this secretory activity may contribute to the biogenesis of drusen [16,47]. Nonetheless, novel vistas emphasize the autophagy activity in the inner choroid. The removal of secreted material at the inner choroid retinal border strongly depends on the permeability of the Bruch’s membrane and the glymphatic and bloodstream in the CC [12]. We may hypothesize that secretory autophagy does not necessarily lead to drusen formation. Endosomes and lysosomes may be released independently of autophagosomes via fatty acid binding protein 4 (FABP4) [152]. FABP4 may shuttle through lysosomes and endosomes toward the extracellular space. This process is independent of the autophagy secretion since it also occurs in ATG5 knock-out conditions [152]. It is likely that, when autophagy is impaired, a higher number of fatty acids is released through such a non-conventional, non-autophagy-dependent secretory pathway, thus contributing to enriching the drusen with lipids. Secretory lysosomes are described within RPE cells [153]. Conversely, when autophagy is still working, a physiological secretory autophagy takes place, which is followed by the removal of aggregates from the extracellular space through the glymphatic and bloodstream in the inner choroid [12]. Thus, when the autophagy pathway still possesses a residual activity, the clearance through the inner choroid may compensate for the accumulation of autophagy-dependent extracellular release. In line with this, the autophagy status within pericytes and endothelial cells of the CC is critical for promoting the bloodstream and glymphatic flow, which is expected to remove extracellular aggregates at the choroid–retinal border [12]. Since the activity of RNVU crucially depends on autophagy [12], one may expect that when autophagy failure extends to the inner choroid, there is an impairment of both the glymphatic and the bloodstream, which impedes the removal of extracellular aggregates from the inner choroid. In this way, released cargoes are persisting beyond the retina with no chance of being removed. Both Bruch’s membrane [98] and pericytes/endothelial cells of the inner choroid are affected concerning shape and metabolism by the autophagy status in the course of AMD. For instance, Torisu et al. [154] demonstrated that a physiological autophagy flux is needed in order to maintain vascular lipid homeostasis. Endothelial cells are damaged by an excess of lipids when autophagy activity is decreased (low expression of autophagy gene ATG 7), which impairs the clearing effect of the inner choroid circulation.

## 8. Do Drusen Act as Innocent Bystanders or Effective Toxic Aggregates That Impair the Visual Process?

The focus on drusen as a core pathological finding for defining AMD led to the concept that these extracellular aggregates are mostly detrimental and produce the classic alterations of the visual process in AMD. Such a concept fits well with the impairment of visual acuity in the presence of drusen underlying the macular region of the retina, the loss of contrast, and, specifically, the occurrence of metamorphopsia. The latter consists of visual distortion, where horizontal and/or vertical lines in the visual field are perceived as wavy or not perceived at all [155,156]. At an early stage, metamorphopsia is perceived following eye movement when the eyes try to balance an object in the visual field, which is sensed by two retinas owing to a different planar arrangement [157,158,159]. This leads to automatic compensatory eye movements in an effort to stabilize such an unbalanced vision. Metamorphopsia may be produced by the sudden and significant variation in the planar arrangement of the retina [160]. When drusen underlie the macular pigment epithelium, close parts of the macula are arranged stochastically compared with the physiological planar geometry. The underlying drusen lift the retinal surface compared with retinal areas where drusen are absent. The macular thickness is modified by the presence of drusen, and it can be measured to assess the severity of AMD. These mechanical alterations of retinal planar geometry are routinely considered a typical detrimental effect induced by the presence of drusen. Again, the mechanical stress exerted by the material compressed between the outer retina and inner choroid was considered to affect the physiology of visual perception [160,161]. Thus, mechanisms that produce a loss of visual acuity in AMD patients are routinely attributed to a loss of retinal planarity and a loss of foveal cones, which also suppresses contrast sensitivity. In line with such a correlation between the amount of drusen and occurrence and severity of metamorphopsia, we previously suggested measuring the drusenoid area (or even better, the drusenoid volume) [162,163] to express AMD severity, since this highly correlates with metamorphopsia compared with drusen number or drusen size both in wet and dry AMD [13,164,165]. These concepts, which appear to be grounded in common sense and the occurrence of a correlation between disease severity and the amount of drusenoid areas, are strikingly challenged by extreme conditions. There are subjects where visual impairment is severe with only a slight alteration in the planar pattern of the retina or, vice versa, an extended drusenoid area occurs concomitantly with preserved visual acuity, contrast sensitivity, and a lack of metamorphopsia. Indeed, the loss of autophagy per se alters the visual processing in the retina [83,84], while the occurrence of drusen as physical entities may not necessarily be detrimental [12,166]. Even when ruling out extreme conditions, a marked discrepancy is measured between the total drusenoid area within the macular region and the onset of visual symptoms in AMD patients [166]. Although mechanisms that produce a loss of visual acuity and contrast sensitivity are routinely considered to rely on the loss of retinal planarity and loss of foveal cones [73,167,168,169], a discrepancy exists. This led us to scrutinize the classic hypothesis by postulating that drusen and visual deterioration develop as two separate and independent consequences of similar upstream biochemical alterations. The severity of drusen and the severity of clinical symptoms in AMD are often associated [170,171], although it remains hypothetical that drusen may produce visual deterioration. In a previous study, we reported the lack of a tight correlation between drusen and visual impairment in a group of 20 AMD patients [166]. In detail, despite the occurrence of drusen, none of these patients experienced AMD-related visual alterations. The near BCVA was 99.05% ± 0.01, the far BCVA was 102.55% ± 0.02, and the Amsler test score was 10 ± 0. In these patients with a significant and severe drusenoid area, no decline in visual function was detected. We may suppose that the autophagy impairment, which occurs in AMD, produces both visual impairment and drusen formation, although there is no direct causal relationship between these events. We produced updated evidence that the impairment of canonical autophagy fosters secretory autophagy, which appears as a determinant step in forming extracellular aggregates (including both drusen and pseudodrusen). However, it remains to be clearly established whether the toxicity of this material produces a loss of visual acuity. A paradoxical hypothesis needs to be considered as well. This consists of the segregation of compounds that exert a toxic effect. Thus, chemical species within drusen may be attenuated in their toxic potential for the RPE when being extruded and segregated within extracellular inclusions. This is likely considering the amounts of toxic chemicals, which move out presumably alleviating the RPE from a chronic deleterious effect. In this context, the effects produced by drusen aggregates may likely not play a relevant role in AMD compared with the toxicity exerted by cytosolic-free, oxidized molecules or damaged mitochondria when they are not yet agglomerated within extracellular inclusions. Thus, inclusions would represent a strategy to trap toxic species and convert them into inert aggregates. In line with this, AMD symptoms seem to be more the consequence of an altered metabolism produced by oxidized structures within the cells of the inner choroid and outer retina, focusing on RPE, rather than the mechanical impairment induced by inclusions or aggregates underlying the retina. This is consistent with the hypothesis that a biochemical defect, which is caused by defective autophagy, is more relevant to the impairment of visual processing rather than the deleterious effects on vision produced by inclusions or aggregates. Within this frame, the dysfunction or a loss of photoreceptor activity may be due to freely diffusible toxic species from RPE rather than underlying drusen deposits. Metamorphopsia and loss of visual acuity may occur as a functional loss of autophagy-dependent biochemical steps rather than being due to damage, depending on the presence of drusen. The latter may act as innocent bystanders and a pathological hallmark, simply witnessing autophagy failure [85,172]. This concept is partially expressed by the conclusions by Jones et al. [173], when they stated that in AMD, alterations within RPE cells rather than the amounts of drusen really predict the progression of the loss of visual acuity. Thus, when autophagy is suppressed within RNVU, it is very likely that an impairment of visual acuity may occur early on, when the integrity of photoreceptors and retinal planarity is still fully preserved. It is rather the loss of autophagy as a biochemical cascade modulating the turnover of photopigment and regulating crucial steps in the physiology of the outer segment of photoreceptors that is likely to generate a loss of vision. In fact, the massive involvement of an autophagy failure in the physiopathology of AMD is supposed to alter, at first, the turnover of photopigments [172]. In this context, the measurement of the loss of the autophagy status would predict the severity of AMD to produce visual symptoms more than the amount of the drusenoid area [166]. It is likely that a biochemical alteration, which decreases the clearance of oxidizing species, would be directly responsible for the loss of visual acuity while being secondarily related to morphological alterations such as drusen and pseudodrusen aggregates. This is confirmed by recent studies [174,175,176], which found that RPE cells express autophagy-related genes, which are activated in a time- and space-dependent pattern. These genes within RPE cells code for a key protein in the digestion and recycling of phagocytosed photoreceptor-derived components. The timing of the expression of these genes is related to the amount of light and stressful conditions. For instance, Naso et al. [174] found that light exposure quickly induces the expression of a microRNA-211 (miR-211) target responsive Ezrin, stimulating the biogenesis of lysosomes and fostering retinal clearance. Such a quick effect explains the fast response of autophagy in RPE cells to sustain phototransduction. This confirms the sound data obtained by Datta et al. [177], who found that enhanced autophagy within AMD-RPE cells produces a peptide named retinal pigment epithelium-derived factor (RPEDF), which restores the visual cycle, leaving intact retinal aggregates. This is likely to sustain fast metabolic steps promoted by autophagy and known to be critical in visual acuity, such as the biochemical processing of the opsins and other components of the distal photoreceptor [85,177,178]. Remarkably, the pioneer study by Choi et al. [178] showed that autophagy within RPE cells is required to match light-induced ROS production and light-induced visual processing. Most importantly, amber light, which does not possess a significant pro-oxidant effect, is still inducing the breakdown of lipid droplets through autophagy-related lysosomal degradation even when irradiating peripheral cells. This concept provides a tight binding between light and epigenetic regulation of autophagy-related genes beyond the retina [178]. The timing of autophagy effects within RPE is currently conceived as a protective and long-lasting metabolic modulation. Nonetheless, sudden, cyclic pulses of light can produce short-acting (a few seconds) activation of autophagy-related enzymatic steps [175,176]. When light is present, autophagy-related enzymes are expressed, which code for autophagosomes and lysosomes. Light exposure at various wavelengths directly promotes autophagy [179] by activating the expression of more than 20 genes, which tightly depend on the activation of phototransduction [180]. These genes regulate the autophagy flux, such as the fusion of autophagy vacuoles with the lysosomes. An excess of these compartments routinely occurs in healthy RPE cells [98,181,182,183]. Similarly, those genes that promote the phagocytosis of the outer segment of photoreceptors through LC3 (the so-called LAP, LC3-associated phagocytosis) are highly expressed within RPE [184]. The LAP provides a tailored autophagosome for the outer segment of photoreceptors, providing the quick enzymatic catabolism of the disk membranes. The impairment of this visual “phagocytosis” is likely to impact visual acuity long before drusen are detected at OCT. This may explain the fact that an impairment of visual acuity and even metamorphopsia precedes the occurrence of drusen in the clinical course of AMD. Conversely, upon administration of a natural autophagy activator, the recovery of visual acuity is supposed to anticipate and to exceed the disappearance of drusen. The expression of Rubicon and melanoregulin (key inducers of LAP) occurs depending on light exposure [185,186].

## 9. Commonalities Between Retinal and CNS Degeneration

The established connection between the retinal structure and the CNS moves the point of this overview from the eye to the brain, since it is expected that analogous mechanisms may induce retinal and CNS degeneration, such as PD and AD. The connection between visual symptoms and PD was elegantly articulated by Archibald et al. [187]. A recent manuscript emphasizes the common genetic causes for RP and PD [188]. In detail, this study reports the association of mutations in the gene coding for α-synuclein, which is responsible for PD, and the mutation of the gene CNGA1 coding for the protein Cyclic Nucleotide Gated channel subunit alpha 1, which is typically associated with RP. This finding, which has practical significance, also serves as proof of principle to explain the occurrence of visual symptoms in PD [189], which, among non-motor symptoms, remain largely unexplored. In particular, the presence of postural instability and gait disturbance may often be related to abnormal stereopsis in Parkinsonian persons. The loss of stereopsis is mostly evident in those phenotypes of PD, where gait abnormalities and postural instability prevail [189]. A gross anatomical correlation is established concerning macular thickness and the occurrence of Parkinsonism and cognitive impairment [190]. In detail, the macular thickness is significantly greater in PD patients than in control patients. This difference is amplified when a concomitant impairment in cognitive function is present. Conversely, macular thickness possesses a significant association with both motor and non-motor PD symptoms and cognitive impairment [190]. The specific pathology described in AMD is very similar to that occurring in central degenerative disorders. This is the case in the occurrence of both intra- and extracellular protein aggregates and their ambiguous significance, both as detrimental factors or rather compensatory reactions [191,192,193,194]. Apart from the natural mechanisms leading to PD and AD, most neurodegenerative disorders affecting specific CNS areas are featured by neuronal inclusions where vitamin A is shown to interact with α-synuclein [195]. There is at present a strong debate on whether these neuronal inclusions may have a detrimental or compensatory role. This is replicated in experimental models ranging from acute neurodegeneration in epilepsy [196] to the neuronal aggregates produced by drugs of abuse [192,197] or the damage induced by Parkinsonism-inducing neurotoxin [198]. The mechanisms involved in neuronal damage in CNS acute and chronic degeneration appear to be grounded on a compensatory recruitment of the autophagy pathway, just like the process leading to the early inclusions observed within RPE cells in AMD. Again, a compensatory activation of secretory autophagy appears to produce the occurrence of β-amyloid-positive drusen in AMD in a way that significantly overlaps with the occurrence of β-amyloid-positive drusen in AD. This is confirmed by the high overlap between these disorders [199]. These Authors indicate a correlation between the occurrence of AD and AMD or other ocular degenerative diseases to identify common pathogenic pathways in the search for novel therapeutic strategies. Similarly, a combined staining and processing for the β-Amyloid Precursor Protein (βAPP) exists in AMD and AD [200,201]. These commonalities extend to other proteins, such as the positive staining for the *tau* protein, which leads to the hypothesis that shared molecular pathways exist between AMD and tauopathies in central degenerative disorders [202]. These shared molecular mechanisms are highly suggestive of a final common pathway, where both cell pathology and neurobiology of disease converge in the retina and central CNS areas. One may consider that, despite being concomitant, the protein alterations may have different effects in AMD and CNS degenerative disorders. For instance, when looking at the role of APO-E in AD and AMD, the protein is implicated in both disorders, although the significance appears to be opposite [203]. Similar pathogenic mechanisms are postulated for PD and AMD. A very recent study by Suimon et al. [204] implicated common roles in the pathogenesis of AMD and PD for the leucine-rich repeat kinase 2 (LRRK2) and α-synuclein. This evidence partly explains the significant association between the occurrence of PD and AMD as reported in a recent meta-analysis [205] and a similar strong association between AMD and the occurrence of AD [206].

## 10. Interaction Between Autophagy and Additional Mechanisms Involved in AMD

The impact of autophagy within AMD is strongly connected with both reactive oxygen species and inflammation, which represent detrimental factors in sustaining retinal integrity and promoting AMD.

The relevance of autophagy to sustain retinal integrity and counteract AMD needs to be reconsidered in a wider scenario where additional detrimental effects play a significant role in altering retinal integrity and fostering AMD onset and progression. This is the case of an abundant amount of reactive oxygen species (ROS), which are massively produced within RPE cells. In the macular region of the retina, where cones are densely packed, a great amount of blue light is processed, which corresponds to the wavelength most detrimental to generate ROS [207,208,209,210]. This wavelength has a strong impact on the outer cone segment and the surrounding RPE cell, which includes the cones between the its processes. The relevance of light-derived ROS is consistent with the fact that the outer retina and mostly the RPE are primarily affected in the course of AMD [16,176,211,212]. Thus, a major role of RPE in sustaining retinal integrity consists in counteracting ROS, which constantly engage cell viability in the outer retina, as happens during direct exposure of the macula to natural light. In this frame, RPE cells are driven by light stimulation to develop a high rate of oxidative metabolism [213]. This increases within RPE during the cycle of blue light stimulation, which generates a strong amount of energy and generates light-sensitive molecular species. In turn, this further magnifies the high oxygen demand, mostly in the outer retina at the level of RPE. This generates a site-specific pro-oxidant environment, which partly explains why RPE is so prone to early degeneration in AMD. The high oxidative metabolic activity of RPE is responsible for producing an excess of oxidized chemical species. This impacts the structure of proteins, lipids, and sugars, which are converted into highly toxic metabolites. This requires a massive autophagy engagement in order to mitigate the effects of oxidized molecules and oxidized mitochondria. These include those naturally occurring within RPE cells and those being phagocytized from the adjacent cones, along with buffering glutamate and reducing all trans retinoic acid into 11-cis-retinal [16,184,214]. Therefore, intense autophagy activity even in baseline conditions in healthy subjects is required within RPE cells to preserve integrity [76,215,216,217,218].

The relevance of light-derived ROS explains why, even in healthy subjects, and in baseline conditions, the autophagy flux within RPE is measured to be more intense within outer compared with inner retinal layers [175,176]. The significance of high amounts of retinal autophagy at the inner choroid outer retinal border, besides ROS, is bound to other phenomena such as inflammation. In keeping with multiple facets of retinal autophagy beyond from protecting against retinal oxidation a recent study by Wang et al. [211] demonstrated that RPE cells produce nucleotide-binding oligomerization domain (NOD)-like receptor X1 (NLRX1), an autophagy inducer, which counteracts retinal oxidative damage while suppressing inflammation. In fact, ROS triggers the formation of pro-inflammatory compounds in the retina such as interleukin 1beta (IL-1 beta), tumor necrosis factor alfa (TNF-alpha), IL-6, and macrophage proteine-1 (MCP-1) [211]. Overexpression of NLRX1 within RPE cells reverts autophagy inhibition and suppresses the levels of ROS and inflammasome [211]. Conversely, knocking down NLXR1 inhibits autophagy and activates the inflammasome, which sustains AMD [211]. In fact, activated inflammasome at the inner choroid outer retinal border is seminal to fostering permeability of blood vessels within choriocapillaris and to generate angiogenesis, which characterizes the shift between dry and wet AMD.

## 11. Implications for Therapeutic Strategies

The relevance of autophagy per se and in combination with concomitant mechanisms acting in the course of retinal degeneration suggested alternative and innovative strategies to cure AMD. This is still at an experimental level, although some reports indicate that autophagy activation may be induced either with specific chemical compounds (drugs and/or phytochemicals) or physical agents such as specific wavelengths and even sound pulses [219]. Novel approaches based on gene therapy may be designed to be tailored to each patient’s disease phenotype. This includes the conditional expression of specific autophagy genes within RPE cells in the course of retinal degeneration. In line with this, it is intriguing that autophagy genes may be specifically induced by selective wavelengths [220,221,222]. For instance, amber light, which is a wavelength of roughly 590 nm, is a powerful autophagy inducer, and it has been suggested, along with red and infra-red light, in specific treatment protocols known as photobiomodulation (PBM) in human patients. These treatments are grounded in solid experimental findings. These wavelengths converge on similar cell steps of the autophagy machinery. This contrasts with the deleterious effects of blue light, which overwhelms autophagy and produces RPE pathology. Amber light activates multiple autophagy steps and increases autophagy-related proteins. A few msec following amber light exposure, LC3I is lipidated into LC3II, which starts the autophagy flux. This is concomitant with the increased expression by amber light of Atg5, as another early autophagy inducer. Amber light sustains autophagy progression by extending downstream to stimulate late steps of the autophagy pathway. This is witnessed by the merging of autophagosomes with lysosomes and later on by lysosomal enzyme degradation. How light may translate into a chemical activation of a metabolic pathway remains to be fully established. Nonetheless, it is demonstrated that amber light interacts with the complex leupeptin/NH4Cl, which produces a baseline inhibition of lysosomal clearance. The interaction of amber light removes such an inhibition, thus releasing autophagy in a quick time frame. As a consequence, several stagnant, potentially toxic substrates are cleared by pulses of amber light, which may counteract the course of retinal degeneration [178]. Similar to amber light, red light exerts a powerful antioxidant effect, which is also mediated by the enhanced expression of heat shock protein 70 (HSP 70) acting as a chaperone protein. Autophagy activation induced by red light may lead to the remove of key detrimental proteins, lipids and sugars such as misfolded tau [223]. Such an increase in autophagy-related proteins induced by both red and amber light was evidenced by an augmentation of both autophagosomes and lysosomes [224,225].

The powerful effects of phytochemicals as autophagy activators have generated a number of clinical reports suggesting the potential use of these plant-derived agents to treat AMD.

Several compounds were recently suggested to produce a significant therapeutic effect in AMD. Among them, a special emphasis was placed on naturally occurring molecules derived from plant extracts and named phytochemicals. These were suggested based on empirical evidence and the experimental data showing a strong antioxidant effect along with the ability to act as powerful autophagy inducers and stimulate the biogenesis of mitochondria [9,16,226]. These novel therapeutic options encompass the main mechanisms known to be involved in AMD. The efficacy of phytochemicals in experimental settings is based both on the direct antioxidant and pro-autophagic effects in the metabolism of the cell and on altering gene expression through epigenetic effects, which promote the synthesis of antioxidant and pro-autophagy proteins [227,228,229,230].

These concepts led to challenging phytochemicals at the clinical and experimental level in several retinal disorders, even beyond AMD, including systemic disease affecting retinal integrity, such as diabetes [231,232,233]. The powerful antioxidant effects of phytochemicals suggest that the retinal site where blue light produces most oxidative damage, which corresponds to the RPE and the inner choroid/outer retinal border, is supposed to benefit from most of their therapeutic effects. This is why most of the data concerning the therapeutic potential of phytochemicals are related to AMD [9]. This is strengthened by the findings that these compounds showed additional effects, being able to suppress the genesis of new vessels, which is critical in the development of wet AMD.

The suppression of vascular endothelial growth, along with autophagy stimulation and antioxidant activities, is promising in the long-term treatment of AMD. This is reported for several compounds reviewed by Bosch-Morell et al. [234] such as saffron, ginkgo, bilberry and blueberry, curcuma, carotenoids, polyphenols and vitamins C and E. Clinical data indicate that administration of various phytochemicals in AMD patients, when carried out by combining bilberry, lutein and resveratrol repeated daily for several months produces a suppression of the drusenoid area [9,166] placed at the choroid-retinal border, which is associated with a significant regression of visual symptoms including metamorphopsia. Most of the evidence reported in the present manuscript indicates that the pathogenesis of AMD recruits an excess of reactive oxygen species, autophagy suppression and angiogenesis, which occurs profusely according to abnormal topography. This configure the use of specific phytochemicals as a novel therapeutic option to improve the specific antiangiogenic effects of drugs being used in AMD [10,235,236,237].

A critical issue concerning phytochemicals is the uncertain and unpredictable bioavailability of these compounds administered systemically to reach the retinal tissue [238]. This is why novel formulations are under study to assess the potential use of nanoparticles and drug combinations [237,239].

## 12. Conclusions

The present manuscript analyzed the current evidence on the various roles of autophagy in AMD. A specific emphasis is placed on different kinds of autophagy, such as lipophagy, in modulating retinal integrity. Despite the relevant role of lipids, including cholesterol, which has been known for decades in the pathology of AMD [80,82], recent studies emphasized the role of altered lipophagy compared with disrupted protein clearance in the neurobiology and pathology of AMD. Nowadays, we are aware that, despite immunostaining of drusen is based on the presence of specific proteins, drusen composition is mostly made up of lipids, which characterize the metabolic dysfunction of the RPE cells. The altered lipophagy, along with impaired glycophagy, has a strong impact on mitochondrial activity. This mostly concerns the suppression of mitochondrial biogenesis, which is impaired to a greater extent compared with mitophagy. The fine analysis of altered autophagy steps in the course of AMD greatly overlaps with the effects induced by autophagy inhibition produced in RPE cells. This witnesses a specific role of autophagy in the retina, which is involved mostly in recycling those structures involved in removing lipid membranes, acting as a platform for visual transduction. Drusen composition is greatly enriched with photoreceptor outer segments, which constantly engage the RPE cells in their physiological activity.

## Figures and Tables

**Table 1:** Relevant proteins in lipophagy.

Proteins	Effects	References
**Vps4**	Regulates transport within multivesicular bodies	[79]
**MAP1LC3/LC3 (microtubule-associated protein 1 light chain 3)-associated phagocytosis (LAP)**	Recycles the actual platform for phototransduction	[83,84]
**ATRA (all trans retinoic acid)**	Directlystimulates autophagywithin RPE cells	[88]
**Lipidated LC3 (LC3II)**	Promoteselongation and membrane folding of the nascent autophagy vesicle	[46]
**Apo-E**	Removes lipids from RPE; Shuttles lipids across cell membranes.	[35,98,99]
**Phosphoinositides/Oxysterols** **Synaptojanin 1**	Induce lipophagy; Sustain autophagosomal and endosomal trafficking.	[94,100]
**Ceramide**	Promotes the removal of Beclin1 from the Beclin1/Bcl-2 complex, and promotes the interaction of Beclin1/VPS34/VPS15 forming a complex which is a strong lipophagy stimulator	[102]

**Table 2:** Representative mitochondrial alterations within RPE cells following autophagy inhibition.

	Total Mitochondria	% Healthy Mitochondria	% Altered Mitochondria
**Control** (n = 30 cells)	13.72 ± 0.08	74.84 ± 0.22	25.16 ± 0.40
**3-MA, 10 mM** (n = 30 cells)	7.28 ± 0.02 * p < 0.0001	55.71 ± 0.32 * p < 0.0001	44.29 ± 0.32 * p < 0.0001

* *p* < 0.05 compared with controls (one-way analysis of variance, ANOVA, followed by Scheffè’s post hoc analysis).

## Data Availability

Cell data are available in a repository which will be made available upon request.

## Abbreviations

The following abbreviations are used in this manuscript:

3-MA: 3-Methyladenine; AD: Alzheimer’s disease; AGEs: advanced glycation end-products; Akt2: RAC-beta serine/threonine-protein kinase 2; ALFY: autophagy-linked FYVE protein; AMBRA: activating molecule in Beclin1-regulated autophagy; AMD: age-related macular degeneration; APO-E: apolipoprotein E; ARPE: arising retinal pigment epithelium; ATG: autophagy; ATRA: all-trans-retinoic acid; βAPP: beta-amyloid precursor protein; Bcl-2: B-cell lymphoma-2; BCVA: best corrected visual acuity; BNIP3: Bcl-2 interacting protein 3; CC: choriocapillaris; CNGA1: cyclic nucleotide gated channel subunit alpha 1; CNS: central nervous system; DCT1: divalent cation transporter; ECM: extracellular matrix; EMT: epithelial-mesenchymal-transition; ER: endoplasmic reticulum; FABP4: fatty acid-binding protein 4; GRAP: GRB2-related adapter protein; HSPs: heat shock proteins; IL-1 beta: interleukin 1beta; LAMP-1: lysosome-associated membrane protein 1; LAP: microtubule-associated protein 1 light chain 3-associated phagocytosis; LRRK2: leucine-rich repeat kinase 2; MAP1LC3: microtubule-associated protein 1 light chain 3; MEPT: mesenchymal transition; miR: microRNA; MCP-1: macrophage proteine-1; MPTP: 1-methyl-4-phenyl-1,2,3,6-tetrahydropyridine; mtDNA: mitochondrial DNA; mTOR: mechanistic target of rapamycin; mTORC1/2: mechanistic target of rapamycin isoform complex 1/2; MVBs: multivesicular bodies; NLRX1: NOD-like receptor X1; OCT: optical coherence tomography; PD: Parkinson’s disease; PEA: phosphatidylethanolamine; PGC1-α: peroxisome proliferator-activated receptor gamma coactivator 1-α; PI3KC3: class III phosphatidylinositol 3-kinase; PI3P: phosphatidylinositol 3-phosphate; PINK1: PTEN-induced kinase 1; Poly-Ub: poly-ubiquitin; POS: photoreceptor outer segment; RAGE receptor for AGEs; RNVU: retinal-choroid neurovascular unit; ROS: reactive oxygen species; RP: retinitis pigmentosa; RPE: retinal pigment epithelium; RPEDF: retinal pigment epithelium-derived factor; SKN-1: skinhead-1; SNAP23: Synaptosomal-associated protein 23; STBD1: starch binding domain-containing protein 1; SYTL1: synaptotagmin like 1; TFAM: mitochondrial transcription factor A; TGs: triglycerides; TNF-alpha: tumor necrosis factor alfa; TRIM16: tripartite motif containing 16; Ub: ubiquitin; UDP: uridine diphosphate; VPS: vacuolar protein sorting.
